# Transient receptor potential vanilloid type-1 regulates periodontal disease damage via the PI3K/AKT signaling pathway

**DOI:** 10.22038/IJBMS.2022.62992.13924

**Published:** 2022-05

**Authors:** Xiaolu Xu, Yueheng Li, Zhengyan Yang, Zhi Zhou

**Affiliations:** 1 Stomatological Hospital of Chongqing Medical University, Chongqing, China; 2 Chongqing Key Laboratory of Oral Diseases and Biomedical Sciences, Chongqing, China; 3 Chongqing Municipal Key Laboratory of Oral Biomedical Engineering of Higher Education, Chongqing, China

**Keywords:** Alveolar bone loss, Cytokines, Periodontitis, PI3K/AKT, TRPV1

## Abstract

**Objective(s):**

This study aimed to investigate the function of transient receptor potential vanilloid 1 (TRPV1) in regulating periodontal lesions. In addition, we explored the underlying mechanism of the phosphatidylinositol 3-kinase/protein kinase B (PI3K/AKT) pathway.

**Materials and Methods:**

Lipopolysaccharide (LPS) stimulation of human periodontal ligament cells (HPDLCs) was used to construct a periodontitis cell model, and experimental periodontitis (EP) rats were established by ligation. The mechanism by which TRPV1 regulates periodontitis was further verified by injecting the TRPV1 agonist capsaicin (CPS) and antagonist capsazepine (CPZ) into the gingiva of rats; the alveolar bone losses in each group were measured by stereomicroscopy. Real-time quantitative polymerase chain reaction (qRT-PCR) and Western blotting (WB) were used to research the expression of TRPV1 and proinflammatory cytokines, and WB was performed to test the phosphorylation of PI3K and AKT.

**Results:**

*In vitro *experiments showed that LPS induced the upregulation of TRPV1 and proinflammatory cytokines and promoted the phosphorylation of PI3K and AKT proteins in HPDLCs, which was consistent with their expression in the rat periodontitis model. Moreover,* in vivo* studies indicated that CPZ had anti-inflammatory effects through the PI3K/AKT pathway and inhibited bone loss induced by periodontal ligation in rats, while CPS had the opposite effect.

**Conclusion:**

TRPV1 was involved in the process of alveolar bone defects and the inflammatory response in rats with periodontitis induced by ligation. Its mechanism might be related to the phosphorylation of related proteins in the PI3K/AKT signaling pathway.

## Introduction

Chronic periodontitis is an inflammatory disease caused by bacterial infection, with extensive clinical, microbiological, and immunological manifestations (1), resulting from interaction between microbial community of the periodontal environment and the host immune-inflammatory response. It’s the main cause of adult tooth loss and seriously damages the health of periodontal tissue. Therefore, the pathogenesis, clinical diagnosis and treatment of periodontitis have been paid more and more attention by scholars at home and abroad. 

At present, the treatment strategies for periodontal diseases are mainly periodontal scaling and root planing, aiming at removing dental plaque. However, these treatments usually ignore the host immune response of periodontitis patients (2, 3). In local inflammation, the interaction between immune cells and immune effectors in oral bacteria triggers autoimmune reactions, resulting in a series of cytokines (4), including interleukin 1β(IL-1β),and tumor necrosis factor α (TNF-α) (5, 6), being continuously released into periodontal tissues, stimulating the production of secondary mediators such as chemokines and prostaglandins, and chronic inflammation is gradually established (3, 7).

TRPV1 is a nonselective cation channel that can be directly or indirectly activated by various physical and chemical factors, such as mechanical stimulation, thermal stimulation, and inflammatory mediators (8, 9), resulting in an influx of divalent cations and the corresponding physiological or pathological reactions (10). Previous studies have shown that TRPV1 is widely expressed in neuronal cells (11–13), but increasing evidence has shown that TRPV1 exists in some nonneuronal tissues, including immune cells such as T lymphocytes (14), macrophages (15), bronchial fibroblasts (16), vascular endothelial cells (17), and renal tubular cells (18). Moreover (19), the upregulation of TRPV1 is associated with many inflammatory diseases, including periodontitis. Sireerat Sooampon, Waranyoo Phoolcharoen *et al.* (20) a nociceptive ion channel receptor, by capsaicin led to the up-regulation of the osteoprotegerin (OPG found that TPRV1 was activated in periodontal ligament cells under thermal stimulation, and Ca^2+^ influx could be used as a secondary messenger to promote the expression of TNF-α. In addition, inhibiting TRPV1 ion channels was found to interfere with the differentiation of periodontal osteoclasts and osteoblasts* in vitro* (21, 22). Therefore, whether TRPV1 can be used as a drug target and an essential auxiliary means of periodontitis treatment is an essential research direction.

Moreover, periodontitis is regulated by a series of complex signaling pathways within the tissue. PI3K / AKT signaling pathway is a classical pathway to regulate inflammation, which is often activated in the development of a variety of inflammatory diseases, including periodontitis. It plays a significant role in the proliferation and apoptosis of periodontal ligament cells, osteoclast differentiation and cytokine secretion by affecting the activity of downstream effectors (23–25). However, whether PI3K/AKT is directly involved in releasing proinflammatory mediators induced by TRPV1 remains unclear and needs to be further clarified. 

In this study, we established periodontitis cells and animal models with the goal of exploring the issues above. It was preliminarily proven that TRPV1 might regulate inflammatory factors through the PI3K/AKT pathway, thereby affecting the occurrence and progression of bone destruction and providing new approaches to the diagnosis and treatment of patients with periodontitis.

## Materials and Methods

***HPDLCs culture and drug treatment***

All experiments were conducted under The Code of Ethics of the World Medical Association, ARRIVE guidelines, The Guide for the Care and Use of Laboratory Animals of the National Research Council, and were compliant with the agreement approved by the Ethics Committee of the School of Stomatology, Chongqing Medical University (NO:2022(LSNo.010)).

Fresh healthy and caries-free premolars of patients aged 10-18 were extracted for orthodontic reasons. Informed consents were obtained from the patients. The periodontal membrane was carefully scraped from the middle 1/3 of the tooth root surface. After digestion in 3 mg/ml type I collagenase (Sigma–Aldrich Company, USA) for 30 min at 37 °C, periodontal ligament tissue was inoculated into a culture flask with α-MEM medium (HyClone, USA) containing 15% fetal bovine serum (Gemini, USA) and 1% penicillin–streptomycin solution (Beyotime, China). The cells were cultured in a humidified atmosphere at 37 °C with 95% air and 5% CO_2_ by the enzymatic tissue block method. Until the cells migrated out of the tissue block, the medium was changed every three days. When the HPDLCs grew to 80% confluence and fused, they were digested with 0.25% trypsin (Sigma–Aldrich, USA) and seeded in a 10 cm petri dish with 10% α-MEM complete medium. Three- to four-generation HPDLCs were inoculated in a six-well plate for 24 h at 2 × 10^4^ cells/well and then grouped into the control group, PBS group (negative control group), and LPS group (periodontitis cell model group). LPS (Sigma–Aldrich, USA) was resuspended in PBS solution (Mengbio, China) until completely dissolved to reach a 1 mg/ml concentration and then added to 10% α-MEM complete medium to dilute it to a final concentration of 10 µg/ml (26–28). The LPS group was incubated with an equal amount of LPS. After incubation with different media for 24 hr, three groups of cells were collected to measure the subsequent indicators.

***Flow cytometry assay***

The third generation of HPDLCs in the logarithmic growth period were digested and adjusted to 1 × 10^6^ cells/ml for further use. Subsequently, primary antibodies (conjugated with secondary antibodies) against CD31, CD45, CD73, CD90, and CD105 (Biolegend, USA) were added to the HPDLCs. The signals of the labeled cells were detected by flow cytometry, and the data were analyzed by FlowJo software.

***Establishment of animal models and grouping administration***

SPF Sprague–Dawley (SD) rats were purchased from the Experimental Animal Center of Chongqing Medical University, placed in sterile rooms designated by the Chongqing Key Laboratory of Oral Diseases and Biomedicine, and provided with free access to water and food by the Animal Health Service Centre.

Construction of the periodontitis model: Male SD rats were anesthetized with isoflurane inhalation, a tip probe was used to separate the gingiva of the first molar, and the gingival sulcus was ligated with sutures (4-0) and orthodontic stainless-steel wires (0.4 mm).

We selected 200–220 g SD rats and divided them into six groups: control group, experimental periodontitis (EP) group, EP + PBS group, EP + capsaicin (CPS) (Abmole, USA) group, EP + capsazepine (CPZ) (Abmole, USA) group, and EP + LY (PI3K/AKT inhibitor LY294002, Abmole, USA) group, with three rats in each group. CPS, CPZ, and LY294002 were dissolved in dimethyl sulfoxide (Sigma, USA) for storage until the above drugs were injected locally and then they were diluted with PBS to a final concentration of 10 µM (17, 19, 21). Immediately after dilution, they were injected into the gingival tissues of the maxillary first molars of the SD rats (200 μl on the buccal and lingual sides) and ligated simultaneously. Drug treatment and model construction continued for four weeks, with a one-day interval of administration and frequent checks of the ligation. If necessary, the ligation was replaced. The experimental rats were euthanized four weeks later, and the free gingiva of the maxillary first molar and residual periodontal tissues (including the teeth) were selected for the subsequent experiments. Each experiment was repeated at least three times, and all operations were performed by the same experimenter.

***Morphological and immunohistochemical analysis of alveolar bone in rats***

The maxillary bones of rats in the control and EP groups were fixed in 4% paraformaldehyde (Biosharp, China) for 24 hr and then decalcified in 10% ethylenediaminetetraacetic acid (EDTA, Solarbio, China) solution for 4-6 weeks until the tissue was softened entirely. After gradient ethanol dehydration, paraffin embedding, sectioning, routine HE staining, and neutral resin sealing, the destruction of the periodontal tissue was imaged on a digital slide scanner.

The paraffin sections were dewaxed, rehydrated, and antigen-repaired in a water bath. Afterward, they were blocked with 3% hydrogen peroxide (H_2_O_2_) solution for 10 min and with goat serum (Bioss, China) at 37 °C for 30 min. After incubation with mouse anti-rat TRPV1 antibody (1:100, Santa Cruz Biotechnology, USA) overnight at 4 °C and anti-mouse secondary antibody (Bioss, China) at room temperature for 1 hr, diaminobenzidine (DAB, Zsbio, China) and hematoxylin (Solarbio, China) staining and neutral resin sealing were applied. Then, the sections were observed and imaged on a slide scanner.

***Detection of alveolar bone loss in rats***

Alveolar bone loss was imaged under a stereomicroscope, and the distance between the cement-enamel junction (CEJ) and the alveolar crest was measured by Zeiss Zen (2012) image analysis software to calculate the alveolar bone absorption value. The specific measurement steps were as follows: After removing the soft tissue under a stereomicroscope, the maxillas of the rats were put into EP tubes filled with 1% methylene blue (Bkmam, China), stained for 10 min, then rinsed with flowing water for 10 min. The angle of view when the buccal tip of the first molar overlapped with the tongue tip was selected for imaging at a total magnification of 16.3. The mesial, central, and distal sites of the buccal and palatal alveolar bone of each tooth were measured, and their mean values were used as the alveolar bone loss values. Each sample was measured three times, taking the average as the final result. The same experimenter performed all of the imaging and measurements.

***Real-time PCR***

The total RNA of HPDLCs and rat gingival tissues was extracted with TRIzol according to the manufacturer’s instructions (Sigma, USA). A Nanodrop 2000 Spectrophotometer (Thermo Scientific, USA) was used to detect the optical density of the extracted RNA at 260 nm and 280 nm, and the concentration and purity were calculated. Reverse transcription was performed using PrimeScript RT Master Mix (Perfect Real Time) (Takara, Japan). A 10 μl reaction volume was used for PCR amplification on a CFX Connect Real-Time System (Bio-Rad, USA), with 5 μl of 2× TB Green Premix Ex Taq II (Takara, Japan), 0.4 μl of upstream primer and 0.4 μl of downstream primer at a concentration of 0.4 μmol/l, 1 μl of DNA template, and 3.2 μl of DEPC water. The primers were synthesized by Takara (Table 1). Quantitative calculation of gene expression followed the 2^-^^△△^^Ct^ method.

***Western blotting***

Total protein was extracted from HPDLCs and rat gingival tissue with RIPA lysis buffer (Beyotime, China) containing the protease inhibitor phenylmethanesulfonyl fluoride (Beyotime, China) and phosphatase inhibitor cocktail (Abmole, USA). Protein concentrations of the samples were detected by a BCA protein quantification kit (Beyotime, China); 30 μg of total protein from each sample was subjected to 10% SDS–PAGE. After transferring the protein in the gel to a polyvinylidene fluoride membrane (Millipore, USA) with a 0.45 μm pore size, nonspecific binding sites on the PVDF membranes were blocked with BSA at room temperature for 2 hr. Subsequently, the membranes were incubated with primary antibodies against TRPV1 (1:500), PI3K (1:1000, Affinity Biosciences, USA), phospho-PI3K (1:1000, Affinity Biosciences, USA), AKT (1:1000, Affinity Biosciences, USA), phospho-AKT (1:1000, Affinity Biosciences, USA), IL-1β (1:1000, ZenBioScience, China), and GAPDH (1:1000, CST, USA) overnight at 4 °C, followed by an anti-rabbit or anti-mouse HRP-labeled secondary antibody (Bioss, China) at room temperature for 90 min. Finally, the signal was revealed with BeyoECL Plus solution (Beyotime), and the gray values of the image were analyzed by ImageJ software.

***Statistical analysis***

GraphPad Prism 7.0 (GraphPad Software, USA) and SPSS 23 (IBM, USA) were used for data processing and statistical analysis. The data results of three independent repeated experiments are shown as the mean ± SD. Student’s t-test was used to compare two mean values. Differences were statistically significant when *P*<0.05. All experiments were repeated at least three times independently.

## Results

***Isolation, culture, and identification of HPDLCs***

**Figure 1:**
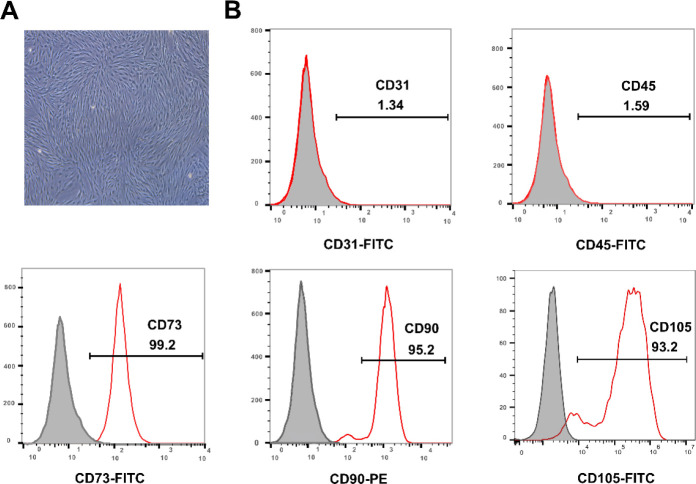
Isolation, culture, and identification of HPDLCs. (A) Morphology of the third generation of HPDLCs (×40). (B) Expression of cell surface molecules by flow cytometry: CD31 (1.34%) and CD45 (1.59%), CD73 (99.2%), CD90 (95.2%), and CD105 (93.2%)

HPDLCs were cultured by enzymolysis of the tissue block, and they showed long spindle shapes when passaged to the third generation (Figure 1A). The surface antigen of the HPDLCs was detected by flow cytometry, demonstrating high expression of the mesenchymal stem cell (MSC) markers CD73, CD90, and CD105, while the markers CD31 and CD45 of hematopoietic stem cells were negatively expressed (Figure 1B).

***LPS upregulated TRPV1 expression and induced phosphorylation of PI3K and AKT in HPDLCs***

**Figure 2:**
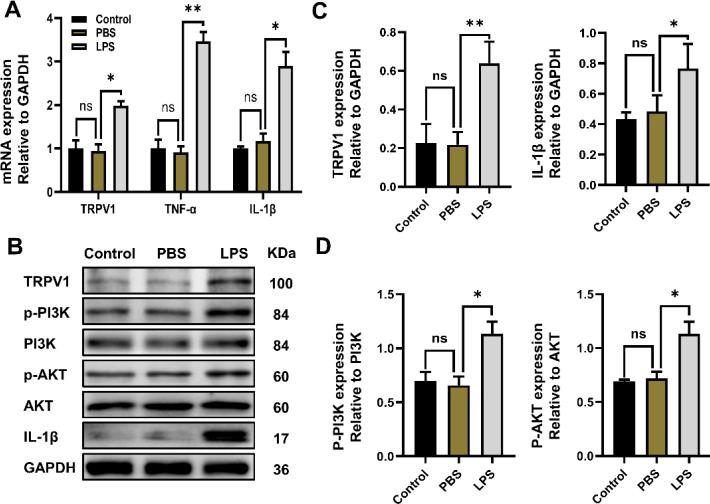
Effect of inflammatory stimulation on transient receptor potential vanilloid 1 (TRPV1) and PI3K/AKT *in vitro*. (A) The mRNA levels of TRPV1, TNF-α, and IL-1β in HPDLCs of the LPS (lipopolysaccharide) PBS and control groups were detected by real-time PCR. (B) Western blotting was used to detect the expression levels of TRPV1, PI3K, phospho-PI3K, Akt, phospho-Akt, IL-1β, and GAPDH. (C) Analysis of the protein expression levels of TRPV1 and IL-1β relative to GAPDH. (D) Analysis of PI3K and AKT phosphorylation levels. The data are expressed as the mean ± SD of three independent experiments. *P<0.05, * *P<0.01, * * * *P*<0.001, ns = no significant difference

To study the expression of TRPV1 ion channels and the PI3K/AKT signaling pathway in HPDLCs under inflammatory conditions, we established LPS PBS and control groups to detect the differential expression of related molecules. The periodontitis cell model was established by LPS stimulation of HPDLCs, and the increased expression of inflammatory factors proved the successful establishment of the model. TRPV1 mRNA and protein expression in the LPS group was significantly higher than that of the control and PBS groups, and the ratio of phospho-PI3K/PI3K and phospho-AKT/AKT was also increased (Figure 2).

***Ligation induced periodontal tissue injury in rats; LY294002 alleviated the above reaction***

**Figure 3:**
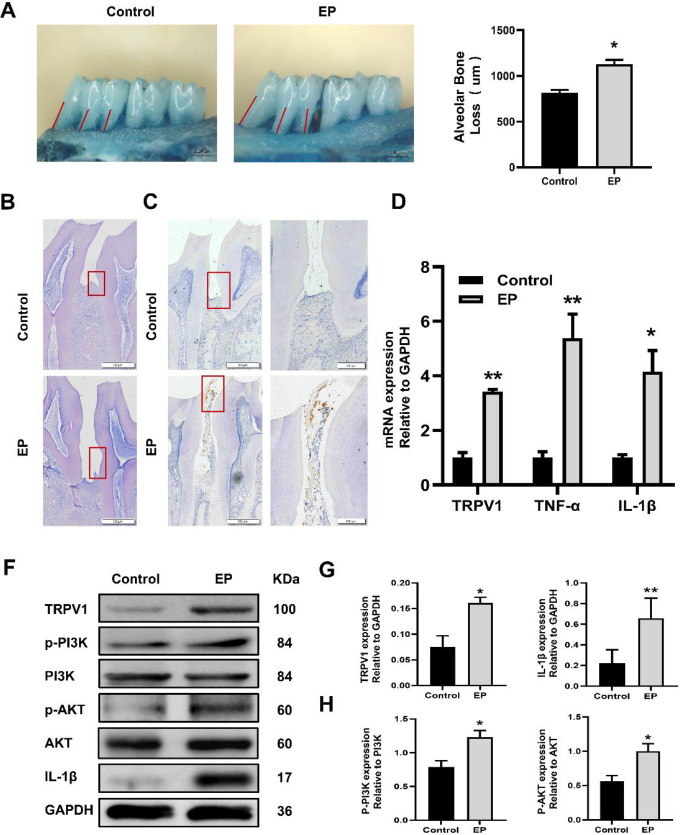
The experimental periodontitis (EP) rat model was established by ligation. (A) The alveolar bone loss of rats in the EP and control groups was imaged by stereomicroscopy, and statistical analysis was performed. Scale bars = 1000 μm. (B) Periodontal tissue HE staining. Box areas represent bone resorption. Scale bars = 500 μm. (C) Immunohistochemical staining of transient receptor potential vanilloid 1 (TRPV1) in periodontal tissues. The right figure is an enlarged image of the left selected area. Scale bars: left= 500 μm; right = 200 μm. (D) The mRNA levels of TRPV1, TNF-α and IL-1β were detected by real-time PCR, with GAPDH as a control. (E) Western blotting was used to detect the expression levels of TRPV1, PI3K, phospho-PI3K, Akt, phospho-Akt, and IL-1β. (F) Analysis of TRPV1 and IL-1β protein expression levels, with GAPDH as a control. (G) Analysis of PI3K and AKT phosphorylation levels. The data are expressed as the mean ± standard deviation of three independent experiments. * *P*<0.05, * * *P*< 0.01, * * * *P*< 0.001, ns = no significant difference

**Figure 4:**
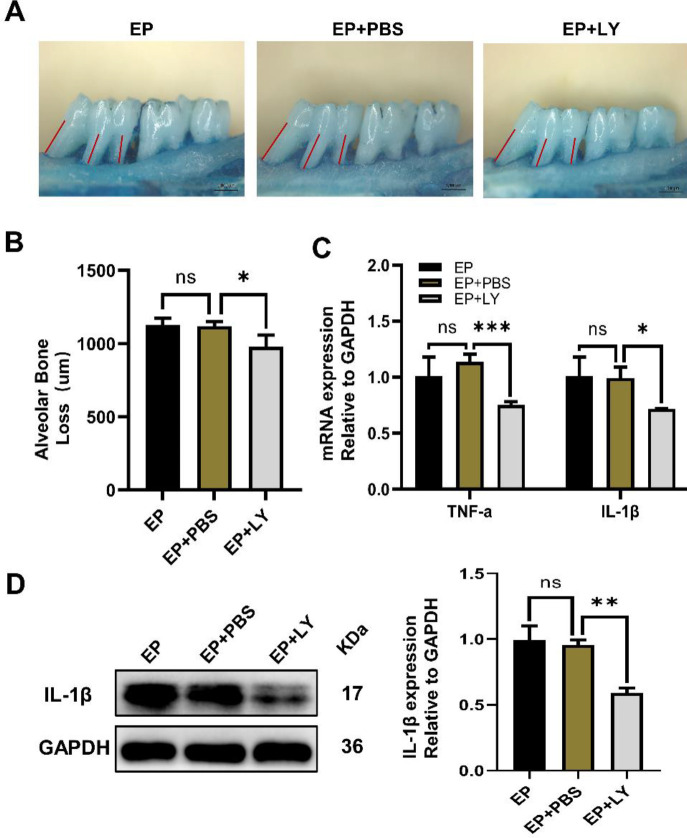
SD rats were given 10 µM PI3K/AKT inhibitor LY294002 (LY) by periodontal injection and subjected to ligation for 4 weeks. (A) The alveolar bone loss of rats in the EP (experimental periodontitis) EP + PBS and EP + LY groups was imaged by stereomicroscopy. Scale bars = 1000 μm. (B) Statistical analysis of the alveolar bone loss of three groups. (C) The mRNA levels of TNF-α and IL-1β were detected by real-time PCR, and GAPDH was used as a control. (D) Western blotting was used to detect the protein expression level of IL-1β relative to GAPDH, and grayscale value analysis was performed. The data are expressed as the mean ± SD of three independent experiments. *P<0.05, * * *P*<0.01, * * *P<0.001, ns = no significant difference

*In vivo*, we constructed a ligation-induced experimental periodontitis rat model. Hematoxylin-eosin (HE) staining results showed that the EP group showed more disordered collagen bundles and more obvious attachment loss than the control group, which confirmed the successful establishment of the periodontal disease model *in vivo*. The upregulation of mRNA and protein of inflammatory factors in the gingival tissue of the EP group further verified the results above. In addition, the qRT-PCR immunohistochemistry and WB results showed that the expression of TRPV1 in the EP group was significantly higher than that in the control group. WB results showed that the PI3K and AKT protein phosphorylation levels were significantly increased in the EP group, and the alveolar bone loss was elevated under a stereomicroscope. The difference was statistically meaningful (*P*<0.05) (Figure 3). To investigate the impact of PI3K/AKT on periodontal tissue in rats, we used the PI3K/AKT-specific inhibitor LY294002. The results showed that LY294002 reduced the inflammatory response of periodontal tissue caused by ligation and alleviated periodontal damage (Figure 4).

***TRPV1 regulated periodontal injury in rats via the PI3K/AKT signaling pathway***

**Figure 5:**
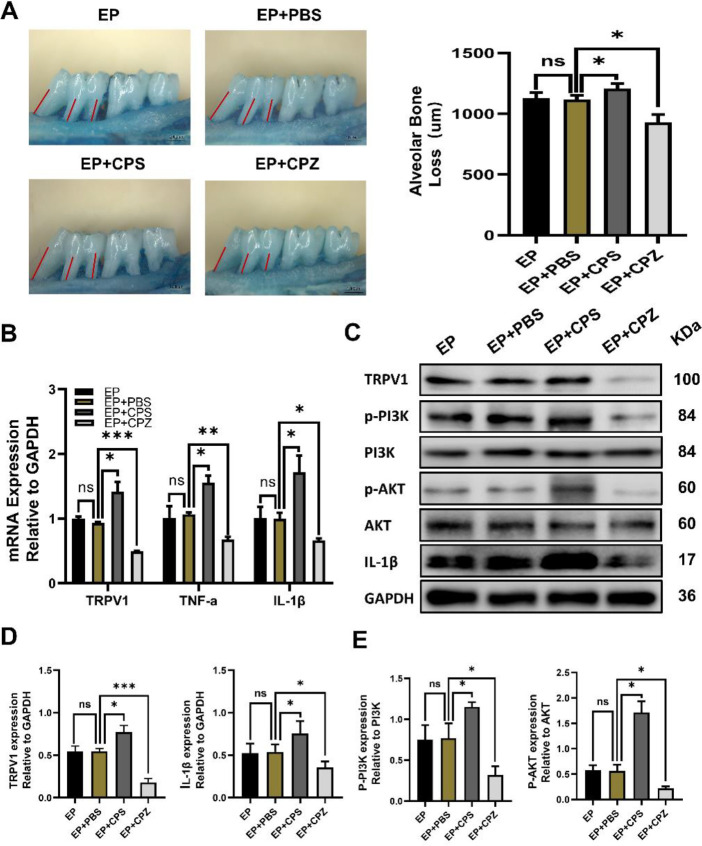
Transient receptor potential vanilloid 1 (TRPV1) positively regulated the secretion of proinflammatory cytokines and aggravated periodontal tissue damage, and the PI3K/AKT signaling pathway was involved. (A) The alveolar bone loss in the EP (experimental periodontitis), EP+PBS, EP+CPS (TRPV1 agonist capsaicin) and EP+CPZ (TRPV1 antagonist capsazepine) groups was measured by stereomicroscopy and statistically analyzed. Scale bars = 1000 μm (B) The mRNA levels of TRPV1, TNF-α and IL-1β were detected by real-time PCR with GAPDH as a control. (C) Western blotting was used to detect the expression levels of TRPV1, PI3K, phospho-PI3K, Akt, phospho-Akt, and IL-1β. (D) Analysis of the protein expression levels of TRPV1 and IL-1β relative to GAPDH. (E) PI3K and AKT phosphorylation level analysis. The data are expressed as the mean ± standard deviation of three independent experiments. * *P*< 0.05, * * *P*<0.01, * * * *P*<0.001, ns = no significant difference

To explore whether the PI3K/AKT pathway is involved in the process of TRPV1 regulating the periodontal inflammatory response in rats, we used the TRPV1-specific agonist CPS and antagonist CPZ to construct the TRPV1 overexpression and inhibition groups. The expression of key molecules p-PI3K, PI3K, p-AKT, and AKT in the PI3K/AKT pathway and inflammatory factors in the gingival tissues of each group were detected. The loss of alveolar bone in each group was observed by stereomicroscop. The results showed that under the effect of CPS, the expression of the proinflammatory factors IL-1β and TNF-α in gingival tissue was increased, alveolar bone destruction was aggravated, and the ratios of phospho-PI3K/PI3K and phospho-AKT/AKT were upregulated, while CPZ had the opposite effect (Figure 5).

**Table 1:** The sequence of primers used in this study for the real-time PCR reaction

**Gene**	**Forward Primer Sequence (5** ^0^ ** to 3** ^0^ **) **	**Reverse Primer Sequence (5** ^0^ ** to 3** ^0^ **)**
**TRPV1(Human)**	**GGCTGTCTTCATCATCCTGCTGCT**	**GTTCTTGCTCTCCTGTGCGATCTTGT**
**TRPV1(Rat)**	**CAGCCAACGCAAGGAGTATG **	**CAAACAAGAACACGAGGTAGACG**
**TNF-α(Human)**	**CTCTFFCCCAFFCAFTCAGA **	**GGCGTTTGGGAAGGTTGGAT**
**TNF-α(Rat)**	**CTTCTCATTCCTGCTCGTGG**	**CCGCTTGGTGGTTTGCTAC**
**IL-1β(Human)**	**GGAGATGACAGTTCAGAAG**	**GTACTGGTGCCGTTTATGC**
**IL-1β(Rat)**	**TCCCAAACAATACCCAAAGAAG**	**ACTATGTCCCGACCATTGCTG**
**GAPDH(Human)**	**TCAAGAAGGTGGTGAAGCAGG **	**AGCGTCAAAGGTGGAGGAGTG**
**GAPDH(Rat)**	**ACAGCAACAGGGTGGTGGAC**	**TTTGAGGGTGCAGCGAACTT**

## Discussion

Periodontitis is a chronic inflammatory disease with periodontal inflammation, attachment loss and alveolar bone resorption caused by dental plaque, and its treatment is the focus of many experts and scholars at home and abroad. Therefore, the successful establishment of a standard periodontitis model is the key to effective research.

First, our experiment relied on LPS to stimulate HPDLCs, establishing a periodontitis model* in vitro*. LPS, derived from Gram-negative bacteria accumulating on the tooth surface, is the main virulence factor causing the immune response of periodontitis hosts (29). It plays a paramount role in periodontal disease and alveolar bone destruction by producing proinflammatory mediators such as IL-1β, IL-6, and TNF-α (30), which are multifunctional cytokines produced by lymphocytes, monocytes, and fibroblasts with extensive biological activities. Most cells can synthesize and secrete inflammatory mediators after stimulation by exogenous antigens or mitogens, causing cell damage and periodontal ligament damage (31–33). Studies have found that blocking the IL-1β receptor can significantly reduce osteoclast formation and bone resorption activity, thereby slowing the destruction of periodontal tissue (4). Moreover, HPDLCs, which are similar to other fibroblasts, are living periodontal cells around the alveolar bone that actively participate in tissue homeostasis and inflammation through proliferation, differentiation, osteogenic induction, and other functional activities (34). In this study, we successfully simulated a periodontitis cell model by LPS stimulation of HPDLCs. In addition, we found that ligation around rat molars could lead to plaque accumulation and eventually periodontal gingival injury and subsequent bone resorption (35). On the above basis, our study successfully applied the previous empirical methods of animal modeling (36) of periodontitis and adopted a double ligation of silk and orthodontic ligation wire. It avoided the problems caused by the silk used by itself easily falling off, and the orthodontic ligation wire used by itself not being manageable to accumulate plaque, ensuring the successful establishment of the periodontitis model. Bone resorption and proinflammatory mediator expression in the periodontitis model were closely related to the accumulation of periodontal bacteria in the ligation area.

TRPV1 is involved in the process of immune activation and the inflammatory response. Studies have found that CD4^+^ and CD8^+^ T lymphocytes mediate the cellular immune response, which is dependent on the Ca2^+^ channels in the cell membrane. TRPV1 can trigger an influx of Ca2^+^ under the action of capsaicin, thereby causing the activation of T cells and triggering the body’s autoimmune response (37). In airway diseases such as asthma and chronic obstructive pulmonary disease, the expression of TRPV1 in bronchial fibroblasts is upregulated in the presence of potential inflammatory stimuli (16). In addition, in patients with atopic dermatitis and advanced osteoarthritis, the TRPV1 receptor also showed increased gene expression (38). This phenomenon also existed in oral inflammatory diseases. Rotpenpian N *et al.* found that the expression of TRPV1 in the TMJ model injected with complete Freund’s adjuvant was more potent than that in normal tissues (39). These findings agree with the present results. In both *in vivo *and *in vitro* inflammatory modeling environments, the expression of TRPV1 was identically increased, suggesting that TRPV1 might positively affect periodontal inflammation and become a new therapeutic target for inflammatory diseases such as periodontitis.

In addition, active excitation of the capsaicin receptor TRPV1 in inflammatory diseases such as pancreatitis, nephritis, and pneumonia has been shown to promote inflammation (40, 41). Vigna SR *et al*. (42) reported that the activation of TRPV1 was involved in the occurrence and progression of acute pancreatitis, and the TRPV1 antagonist capsaicin could increase a variety of inflammatory parameters, such as peroxidase activity and pancreatic edema. Additionally, Chien-Lin Lu *et al*. (18) found that hyperfunction of TRPV1 can activate caspase-1, release IL-1β, and trigger tubule cell inflammation, while inhibiting TRPV1 can alleviate the above symptoms. In periodontal disease, N.-L. Avellan (43) reported that the local application of capsaicin to the alveolar mucosa could cause a significant elevation and activation of MMP-8, an essential destructive protease in periodontitis. 

The above studies agreed with our current research results. In our study, the TRPV1 agonist CPS was injected into the periodontal tissue of experimental periodontitis rats, and the level of inflammatory mediators in the tissue was increased, along with periodontal attachment loss. In contrast, CPZ, an antagonist, had a negative regulatory effect. However, previous studies have focused less on the specific mechanism by which TRPV1 regulates the inflammatory response, so our subsequent studies concentrated on this aspect.

The PI3K/AKT pathway is one of the most frequently activated signaling pathways in inflammation, including periodontitis (44, 45). It can regulate various inflammatory factors and chemokines, thus affecting the emergence and development of inflammation. Farrerol, veratric acid, and other drugs were found to inhibit the production of IL-6, IL-8, or osteoclast formation in periodontal tissue by inhibiting the PI3K/AKT signaling pathway (46–48). Therefore, we detected proteins related to the PI3K/AKT pathway in gingival tissues of the EP+CPS and EP+CPZ groups by WB. The results showed that PI3K and AKT protein phosphorylation levels, TRPV1 expression, and periodontal inflammation levels had synergistic effects, suggesting that the PI3K/AKT pathway may be one of the crucial pathways of TRPV1-mediated periodontal inflammation and that it positively affects the process of periodontal tissue damage.

Nevertheless, our experiment was merely a preliminary exploration of the related mechanism and still had many limitations. Upstream events leading to upregulation of TRPV1 in periodontitis were not investigated. In addition, the specific molecular mechanism by which TRPV1 regulates the PI3K/AKT pathway and proinflammatory mediator secretion remains unclear and should be further investigated. Due to the complexity and diversity of periodontitis, the expression level and biological functions of TRPV1 need to be verified in other periodontal tissue cells. At the same time, the relationship between TRPV1 expression and clinicopathological features in patients with periodontitis needs to be studied in more clinical samples.

In summary, TRPV1 is involved in the regulation of periodontal injury, and the PI3K/AKT signaling pathway may play a significant role. Accordingly, TRPV1 has the potential to become a new therapeutic target for periodontitis; inhibition of TRPV1 gene activity may become a new and biological-based treatment for periodontitis, which has broad potential for clinical management of periodontitis. An in-depth study of TRPV1 has prospective and guiding effectiveness for the treatment and prognosis of periodontitis patients.

## Conclusion

Using models of periodontitis *in vitro* and *in vivo,* we tested the hypothesis that TRPV1 was upregulated under periodontal inflammatory conditions. The present findings confirmed that phosphorylation of PI3K/AKT is involved in TRPV1-mediated periodontal inflammation, and the ratio of phospho-PI3K/PI3K and phospho-AKT/AKT had a synergistic effect with the expression of inflammatory factors in gingival tissue. Additional studies are required to explore the function and internal mechanism of TRPV1 in different periodontal-related cells and clinical models.

## Authors’ Contributions

All authors contributed to the research design. XX, YL and ZhY Carried out experiments, analyzed data, made figures, and drafted the manuscript. ZhY and ZhZh Revised the manuscript. All authors approved the final version of the manuscript.

## Conflicts of Interest

All authors claim no conflicts of interest.
